# Supplementary material for: Diagnostic Accuracy of the LabTurbo QuadAIO Common Flu Assay for Detecting Influenza A Virus, Influenza B Virus, RSV, and SARS-CoV-2
Source: Diagnostics (Basel). 2024 Oct 2;14(19):2200. doi: 10.3390/diagnostics14192200 (PMC11475264; doi:10.3390/diagnostics14192200)
Supplement: Supplementary file 1 [file diagnostics-14-02200-s001.zip › diagnostics-3218891-supplementary.pdf]

Supplementary Table 1 Clinical Evaluation of Negative Samples between LabTurbo QuadAIO and Xpert Xpress Assays

Sample ID	All Targets[#]	All Targets[#]	Conformity
	**LabTurbo QuadAIO Assay**	**Xpert Xpress Assay**	Yes/No
**TSGH-300**	No Ct*	No Ct	Yes
**TSGH-301**	No Ct	No Ct	Yes
**TSGH-302**	No Ct	No Ct	Yes
**TSGH-303**	No Ct	No Ct	Yes
**TSGH-304**	No Ct	No Ct	Yes
**TSGH-305**	No Ct	No Ct	Yes
**TSGH-306**	No Ct	No Ct	Yes
**TSGH-307**	No Ct	No Ct	Yes
**TSGH-308**	No Ct	No Ct	Yes
**TSGH-309**	No Ct	No Ct	Yes
**TSGH-310**	No Ct	No Ct	Yes
**TSGH-311**	No Ct	No Ct	Yes
**TSGH-312**	No Ct	No Ct	Yes
**TSGH-313**	No Ct	No Ct	Yes
**TSGH-314**	No Ct	No Ct	Yes
**TSGH-315**	No Ct	No Ct	Yes
**TSGH-316**	No Ct	No Ct	Yes
**TSGH-317**	No Ct	No Ct	Yes
**TSGH-318**	No Ct	No Ct	Yes
**TSGH-319**	No Ct	No Ct	Yes
**TSGH-320**	No Ct	No Ct	Yes
**TSGH-321**	No Ct	No Ct	Yes
**TSGH-322**	No Ct	No Ct	Yes
**TSGH-323**	No Ct	No Ct	Yes
**TSGH-324**	No Ct	No Ct	Yes
**TSGH-325**	No Ct	No Ct	Yes
**TSGH-326**	No Ct	No Ct	Yes
**TSGH-327**	No Ct	No Ct	Yes
**TSGH-328**	No Ct	No Ct	Yes
**TSGH-329**	No Ct	No Ct	Yes
**TSGH-330**	No Ct	No Ct	Yes
**TSGH-331**	No Ct	No Ct	Yes

**TSGH-332**	No Ct	No Ct	Yes
**TSGH-333**	31.79 (RSV)	No Ct	No
**TSGH-334**	No Ct	No Ct	Yes
**TSGH-335**	No Ct	No Ct	Yes
**TSGH-336**	No Ct	No Ct	Yes
**TSGH-337**	No Ct	No Ct	Yes
**TSGH-338**	No Ct	No Ct	Yes
**TSGH-339**	No Ct	No Ct	Yes
**TSGH-340**	No Ct	No Ct	Yes
**TSGH-341**	No Ct	No Ct	Yes
**TSGH-342**	No Ct	No Ct	Yes
**TSGH-343**	No Ct	No Ct	Yes
**TSGH-344**	No Ct	No Ct	Yes
**TSGH-345**	No Ct	No Ct	Yes
**TSGH-346**	No Ct	No Ct	Yes
**TSGH-347**	No Ct	No Ct	Yes
**TSGH-348**	No Ct	No Ct	Yes
**TSGH-349**	No Ct	No Ct	Yes
**TSGH-350**	No Ct	No Ct	Yes
**TSGH-351**	No Ct	No Ct	Yes
**TSGH-352**	No Ct	No Ct	Yes
**TSGH-353**	No Ct	No Ct	Yes
**TSGH-354**	No Ct	No Ct	Yes
**TSGH-355**	No Ct	No Ct	Yes
**TSGH-356**	No Ct	No Ct	Yes
**TSGH-357**	No Ct	No Ct	Yes
**TSGH-358**	No Ct	No Ct	Yes
**TSGH-359**	No Ct	No Ct	Yes
**TSGH-360**	No Ct	No Ct	Yes
**TSGH-361**	No Ct	No Ct	Yes
**TSGH-362**	No Ct	No Ct	Yes
**TSGH-363**	No Ct	No Ct	Yes
**TSGH-364**	No Ct	No Ct	Yes
**TSGH-365**	No Ct	No Ct	Yes
**TSGH-366**	No Ct	No Ct	Yes
**TSGH-367**	No Ct	No Ct	Yes
**TSGH-368**	No Ct	No Ct	Yes

**TSGH-369**	No Ct	No Ct	Yes
**TSGH-370**	No Ct	No Ct	Yes
**TSGH-371**	No Ct	No Ct	Yes
**TSGH-372**	No Ct	No Ct	Yes
**TSGH-373**	No Ct	No Ct	Yes
**TSGH-374**	No Ct	No Ct	Yes
**TSGH-375**	No Ct	No Ct	Yes
**TSGH-376**	No Ct	No Ct	Yes
**TSGH-377**	No Ct	No Ct	Yes
**TSGH-378**	No Ct	No Ct	Yes
**TSGH-379**	No Ct	No Ct	Yes
**TSGH-380**	No Ct	No Ct	Yes
**TSGH-381**	No Ct	No Ct	Yes
**TSGH-382**	No Ct	No Ct	Yes
**TSGH-383**	No Ct	No Ct	Yes
**TSGH-384**	No Ct	No Ct	Yes
**TSGH-385**	No Ct	No Ct	Yes
**TSGH-386**	No Ct	No Ct	Yes
**TSGH-387**	No Ct	No Ct	Yes
**TSGH-388**	No Ct	No Ct	Yes
**TSGH-389**	No Ct	No Ct	Yes
**TSGH-390**	No Ct	No Ct	Yes
**TSGH-391**	No Ct	No Ct	Yes
**TSGH-392**	No Ct	No Ct	Yes
**TSGH-393**	No Ct	No Ct	Yes
**TSGH-394**	No Ct	No Ct	Yes
**TSGH-395**	No Ct	No Ct	Yes
**TSGH-396**	No Ct	No Ct	Yes
**TSGH-397**	No Ct	No Ct	Yes
**TSGH-398**	No Ct	No Ct	Yes
**TSGH-399**	No Ct	No Ct	Yes
**TSGH-400**	No Ct	No Ct	Yes
**TSGH-401**	No Ct	No Ct	Yes
**TSGH-402**	No Ct	No Ct	Yes
**TSGH-403**	No Ct	No Ct	Yes
**TSGH-404**	No Ct	No Ct	Yes
**TSGH-405**	No Ct	No Ct	Yes

**TSGH-406**	No Ct	No Ct	Yes
**TSGH-407**	31.24 (RSV)	No Ct	No
**TSGH-408**	No Ct	No Ct	Yes
**TSGH-409**	No Ct	No Ct	Yes
**TSGH-410**	No Ct	No Ct	Yes
**TSGH-411**	No Ct	No Ct	Yes
**TSGH-412**	No Ct	No Ct	Yes
**TSGH-413**	No Ct	No Ct	Yes
**TSGH-414**	No Ct	No Ct	Yes
**TSGH-415**	No Ct	No Ct	Yes
**TSGH-416**	No Ct	No Ct	Yes
**TSGH-417**	No Ct	No Ct	Yes
**TSGH-418**	No Ct	No Ct	Yes

# All Targets: Influenza A Virus, Influenza B Virus, RSV, and SARS-CoV-2

***No Ct: Indicates that the detection system was unable to detect target pathogen nucleic acid in the sample.**
